# The identification of protein and RNA interactors of the splicing factor Caper in the adult *Drosophila* nervous system

**DOI:** 10.3389/fnmol.2023.1114857

**Published:** 2023-06-23

**Authors:** M. Brandon Titus, Adeline W. Chang, Niko Popitsch, Christopher C. Ebmeier, Jeremy M. Bono, Eugenia C. Olesnicky

**Affiliations:** ^1^Department of Biology, University of Colorado Colorado Springs, Colorado Springs, CO, United States; ^2^Department of Biochemistry and Cell Biology, Max Perutz Labs, University of Vienna, Vienna, Austria; ^3^Department of Biochemistry, University of Colorado Boulder, Boulder, CO, United States

**Keywords:** alternative splicing, RNA-binding proteins, *caper*, RBM39, *Drosophila*, tissue specific function

## Abstract

Post-transcriptional gene regulation is a fundamental mechanism that helps regulate the development and healthy aging of the nervous system. Mutations that disrupt the function of RNA-binding proteins (RBPs), which regulate post-transcriptional gene regulation, have increasingly been implicated in neurological disorders including amyotrophic lateral sclerosis, Fragile X Syndrome, and spinal muscular atrophy. Interestingly, although the majority of RBPs are expressed widely within diverse tissue types, the nervous system is often particularly sensitive to their dysfunction. It is therefore critical to elucidate how aberrant RNA regulation that results from the dysfunction of ubiquitously expressed RBPs leads to tissue specific pathologies that underlie neurological diseases. The highly conserved RBP and alternative splicing factor Caper is widely expressed throughout development and is required for the development of *Drosophila* sensory and motor neurons. Furthermore, *caper* dysfunction results in larval and adult locomotor deficits. Nonetheless, little is known about which proteins interact with Caper, and which RNAs are regulated by Caper. Here we identify proteins that interact with Caper in both neural and muscle tissue, along with neural specific Caper target RNAs. Furthermore, we show that a subset of these Caper-interacting proteins and RNAs genetically interact with *caper* to regulate *Drosophila* gravitaxis behavior.

## Introduction

Alternative splicing is a fundamental gene regulatory mechanism in eukaryotic organisms that increases both transcriptomic and proteomic complexity. Alternative splicing is a highly regulated process that occurs in the majority of the genome. It is estimated that 92–97% of multi-exon encoding genes in humans undergo alternative splicing (Pan et al., 2008; Wang et al., 2008; Scotti and Swanson, 2016). Thus, not surprisingly, when alternative splicing is disrupted it can result in myriad genetic disorders, including blood disorders, neurodegenerative disease, and cancer (Busslinger et al., 1981; Fletcher et al., 2013; Liu and Zack, 2013; Scotti and Swanson, 2016). In fact, it is estimated that 15 to 50% of human genetic diseases are a result of mutations that cause aberrant splicing (Krawczak et al., 1992; Teraoka et al., 1999; Ars et al., 2000; Cáceres and Kornblihtt, 2002; Matlin et al., 2005).

Although aberrant splicing can be the result of a variety of mutations in both cis- and trans-acting elements, there is particular interest in the role that RNA-binding proteins (RBPs), specifically alternative splicing factors, play in the regulation of alternative splicing. Aberrant splicing, due to mutations in RBPs, has been associated with neurological disorders, including the neuromuscular degenerative disease amyotrophic lateral sclerosis (ALS) and autism spectrum disorder (Tazi et al., 2009; Irimia et al., 2014; Scotti and Swanson, 2016). For example, mutations in TDP43, a member of the hnRNP family of RBPs, is associated with nuclear TDP43 protein forming mislocalized TDP43 aggregates in the cytoplasm of individuals with ALS (Tazi et al., 2009). TDP43 has also been shown to repress cryptic splice sites that when aberrantly spliced result in nonsense-mediated decay (Beck et al., 2012; Ling et al., 2015). Furthermore, inclusion of such cryptic exons has been associated with autism spectrum disorder (ASD), underscoring the broad importance of splicing to healthy neuronal function (Irimia et al., 2014; Scotti and Swanson, 2016).

A significant portion of mRNAs that are alternatively spliced undergo developmental-and tissue-specific splicing events (Grosso et al., 2008; Wang et al., 2008; Taliaferro et al., 2011; Sebastian et al., 2013; Badr et al., 2016; Rodriguez et al., 2020). However, despite the tissue-specific splicing observed, very few RBPs and splicing factors are tissue specific, and by contrast are actually widely expressed (Mohr and Hartmann, 2014). The specificity of isoforms required for varying tissues and developmental stages suggests that alternative splicing must be highly regulated, yet little is known how ubiquitously expressed RBPs are able to regulate such distinct splicing events. Alternative splicing is regulated by a combination of interactions between the RNA targets and the RBPs that facilitate splicing (Zhang et al., 2005; Lovci et al., 2013). Furthermore, most of these interactions are relatively low affinity. These low affinity interactions allow for flexibility in sequence specificity, response to relatively small changes in concentration of interacting molecules, and quick exchange of regulatory factors to produce dynamic responses. To overcome the low affinity interactions, the interactions are strengthened by additional interaction between other proteins and ligands (Tazi et al., 2009). This suggests that RBPs and post-transcriptional regulators engage in a mixture of cooperative and competitive interactions to regulate splicing events. Varying concentrations and combinations of splicing regulatory proteins can result in different RNA-protein and protein–protein interactions that result in differential regulation of splicing (Fu and Ares, 2014; Bradley et al., 2015; Brooks et al., 2015). Furthermore, alternative splicing factors can interact with environment-dependent ligands or undergo phosphorylation to further increase specificity of regulation (Xie and Black, 2001; Zhou and Fu, 2013; Fu and Ares, 2014). Interestingly, many splicing regulators have been shown to either regulate their own transcripts or the transcripts of other known splicing regulator proteins (Sureau et al., 2001; Kumar and Lopez, 2005; Salz and Erickson, 2010; Fu and Ares, 2014; Brooks et al., 2015). These interactions make it paramount to identify RBP-interacting molecules to fully understand the tissue- and developmental-specific mechanics that may be employed.

Caper was recently identified as an RBP and alternative splicing factor that is conserved in its role in regulating dendritogenesis in both *Drosophila melanogaster* and *C. elegans* (Olesnicky et al., 2014, 2017). Disruption of *caper* results in aberrant morphology in class IV da sensory neurons, chordotonals, and adult mechanosensory organs in *Drosophila*. Additionally, *caper* hypomorphs show slow climbing speeds compared to control animals in a gravitaxis assay. This phenotype is more severe in males, suggesting Caper may regulate sex-specific splicing (Titus et al., 2021). Caper has also been shown to be localized to the nucleus further supporting its role as an alternative splicing factor (Olesnicky et al., 2017). Interestingly, Caper is also found in cytoplasmic puncta, suggesting that Caper may play RNA regulatory roles beyond splicing (Titus et al., 2021). Furthermore, results from RNA sequencing suggest that Caper may have a strong bias toward regulating 3′ exon usage. This is important as it has been demonstrated that differences in 3′ exon usage often impacts localization of the mRNA (Taliaferro et al., 2016; Olesnicky et al., 2017).

RBM39, the vertebrate ortholog to Caper, has also been identified as an alternative splicing factor that primarily regulates the inclusion or exclusion of cassette exons (Jung et al., 2002). RBM39 is localized to both the nucleus and cytoplasm, and co-localizes with the splicing factor SC35 (Jung et al., 2002; Huang et al., 2012). RBM39 exhibits similar domain architecture as U2AF65, an snRNP in the core splicing machinery, which directly interacts with U2AF. RBM39 has been demonstrated to have a role in several pathways including the transcriptional activation of AP1/Jun and estrogen receptors, and has been associated with colorectal cancers and Ewing sarcoma (Stepanyuk et al., 2016). Murine RBM39 also interacts with another RBP, ZPF106, which is associated with neuromuscular degeneration, suggesting that vertebrate RBM39 orthologs may also have roles in the nervous system. Furthermore, analysis of RNA-seq data shows that genes down-regulated in an RBM39 RNAi background are associated with neurodegenerative diseases (Jung et al., 2002; Anderson et al., 2016).

To better understand the role of *caper* in post-transcriptional regulation, we performed co-immunoprecipitation and RNA immunoprecipitation followed by RNA sequencing to identify putative interacting proteins and RNA targets of Caper in adult nervous tissue. As a comparison, co-immunoprecipitation was also performed in the adult thorax muscles to identify protein interactors that may impart tissue specificity of Caper function. Here we show that while there is overlap of several interacting proteins between both tissues, there is a significant number of unique protein interactions in neuronal tissue compared to muscles. Interestingly, identification of multiple ribosomal proteins, eukaryotic translation initiation factors, and poly-A binding protein (pAbp) in both neural and muscle-enriched tissues suggest that Caper may also have a role in translational regulation. This aligns with the recent discovery that Caper is expressed in the cytoplasm in *Drosophila* larval brains, and that murine RBM39 is found within the cytoplasm (Titus et al., 2021). Our results indicate that Caper regulates over 2000 putative RNA targets in neural tissue that function in neurodevelopment, immune response, and many other processes. However, the molecular function of many of the targets reveals that a vast majority are likely involved in transcriptional, post-transcriptional, and translational regulation. Finally, we performed a candidate-based modifier screen using an adult gravitaxis assay to verify a subset of these protein and RNA interactors.

## Materials and methods

### *Drosophila* strains

The following stocks were obtained from the Bloomington Stock Center: *caper^CC01391^* (Buszczak et al., 2007); *P{GAL4-Hsp70.PB}89–2-1*. *caper^CC01391^* is a hypomorphic allele that was previously characterized (Olesnicky et al., 2017) and animals homozygous for this allele will hereafter be referred to as *caper ^−/−^*. Since the *caper*^CC01391^ hypomorphic mutant allele was created in a *yw* background, *yw* served as the control (Buszczak et al., 2007; Olesnicky et al., 2017). Generation of *UAScaperFLAG* lines was previously described (Titus et al., 2021).

### Immunoprecipitation and immunoblotting

Immunoprecipitation and immunoblotting experiments were performed as previously described (Titus et al., 2021). Immunoprecipitation with FLAG antibody was performed using 50 ul Anti-FLAG® M2 Magnetic Beads (Sigma-Aldrich M8823) incubated with 1,000 ul of lysate for 4 h at 4 °C. Tissue collected from *yw* flies was used as a control for immunoprecipitation performed with FLAG antibody.

### RNA-sequencing protocol

For RIP-Seq, RNA was purified from immunoprecipitations and their respective inputs using TRIzol® Reagent (Invitrogen) extraction. Samples were DNAse I treated and then RNA was isolated using phenol:chloroform:isoamyl and ethanol/sodium acetate precipitation. Samples were sent to University of Colorado Anschutz Medical Campus Genomics Core Facility for library preparation and RNA sequencing.

RNA purity, quantity and integrity was determined with NanoDrop (ThermoFisher Scientific) and TapeStation 4200 (Agilent, CA, United States) analysis prior to RNAseq library preparation. The Tecan Universal Plus Total RNA-Seq with the *Drosophila* probes was used to generate RNA-Seq libraries. Paired-end sequencing reads of 150 nt was generated on NovaSeq 6000 (Illumina, Inc., CA, United States) sequencer at a target depth of 40 million paired-end reads per sample. Raw sequencing reads were de-multiplexed using bcl2fastq.

### RNA-sequencing analysis

Analysis for RNA-sequencing was performed on 20 million to 43 million 150 base-pair paired-end reads. Sequencing adapters were removed, and the reads were quality trimmed with fastp (v0.20.1) and quality was checked with fastqc (v0.11.8). Processed reads were mapped with STAR (v2.7.6a) against the *D. melanogaster* reference genome (Flybase version dmel_r6.36). We observed 53–57% uniquely mapped reads for input samples and 79–89% uniquely mapped reads for pulldown samples. Final read alignments were quality checked and further processed. We furthermore extracted coverage signals from read alignments for manual data inspection in the IGV genome browser. For differential expression analysis, we downloaded gene annotations from flybase and calculated strand-specific count tables with featureCounts (subread v2.0.1) with the following configuration:

featureCounts -T 8 -s 1 -p -Q 20 -t exon -g gene_id -F GTF -a ${gtf} -o feature_counts.tsv ${bams}.

Further data processing was conducted in RStudio (v1.2.5001) using DEseq2 (v1.22.2). From the resulting data, putative interactors were selected by filtering for adjusted *p*-value < 0.05 and log-fold change > 0 from the IP with Caper antibody compared to the input.

### RT-PCR

Three biological replicates of immunoprecipitation were performed using Anti-FLAG® M2 Magnetic Beads (Sigma-Aldrich M8823) on tissue extracted from adult *Drosophila* heads that expressed FLAG-tagged Caper driven by heat-shock. Lysis produced from the heads of *yw* flies were used as a control. RNA was purified from the immunoprecipitation samples and their respective inputs using TRIzol® Reagent (Invitrogen) extraction. Samples were DNAse I treated and then RNA was isolated using phenol:chloroform:isoamyl and ethanol/sodium acetate precipitation. cDNA libraries were created from isolated RNA using a High Capacity RNA-to-cDNA kit (ThermoFisher Scientific, 4,387,406) according to manufacturer protocol. PCR was performed on the cDNA products using One*Taq®* DNA polymerase (New England BioLabs, M0480L) according to manufacturer protocol. Primers were used to identify the presence of RNA for the following genes: *quaking related 58E-1* (*qkr58E-1*), *chickadee* (*chic*), *uncoordinated 115a* (*unc-115a*), *prospero* (*pros*), *longitudinals lacking* (*lola*), zn finger homeodomain 1 (*zfh1*), *acinus* (*acn*), *uncoordinated 115b* (*unc-115b*), *discs large 1* (dlg1), *turtle* (*tutl*), *bruchpilot* (*brp*), and *chronologically inappropriate morphogenesis* (*chinmo*) (Supplementary Table S1). Electrophoresis of PCR products was performed on a 1% agarose gel with ethidium bromide. The gels were imaged using the Azure Biosystems c400.

The RT-PCR products were quantified using FIJI gel analysis tool to measure the intensity of the bands. The intensity of the bands was normalized to their respective inputs and then a T-test was performed on the normalized intensities. *p*-values are listed in Supplementary Table S1.

### Sample preparation for mass spectrometry

Caper affinity purifications were denatured, reduced and alkylated using 5% (w/v) sodium dodecyl sulfate (SDS), 10 mM tris(2-carboxyethylphosphine) (TCEP), 40 mM 2-chloroacetamide, 50 mM Tris–HCl, pH 8.5 with boiling 10 min, then incubated shaking at 1000 rpm at 37°C for 30 min. Proteins were digested using the SP3 method (Hughes et al., 2014). Briefly, 200 μg carboxylate-functionalized speedbeads (Cytiva Life Sciences) were added followed by the addition of acetonitrile to 80% (v/v) inducing binding to the beads. The beads were washed twice with 80% (v/v) ethanol and twice with 100% acetonitrile. Proteins were digested in 50 mM Tris–HCl, pH 8.5, with 0.5 μg Lys-C/Trypsin (Promega) and incubated at 37°C overnight. Tryptic peptides were desalted with the addition of 95% (v/v) acetonitrile binding the peptides back to the beads and washed once with 100% acetonitrile. Peptides were collected from the beads with two elutions of 1% (v/v) trifluoroacetic acid, 3% (v/v) acetonitrile. Cleaned-up peptide were then dried in a speedvac vacuum centrifuge and stored at −20°C until analysis.

### Mass spectrometry analysis

Tryptic peptides were suspended in 3% (v/v) can, 0.1% (v/v) trifluoroacetic acid (TFA) and directly injected onto a reversed-phase C18 1.7 μm, 130 Å, 75 mm X 250 mm M-class column (Waters), using an Ultimate 3000 nanoUPLC (Thermos Scientific). Peptides were eluted at 300 nL/min with a gradient from 2 to 20% ACN in 40 min then to 40% ACN in 5 min and detected using a Q-Exactive HF-X mass spectrometer (Thermo Scientific). Precursor mass spectra (MS1) were acquired at a resolution of 120,000 from 350 to 1,550 m/z with an automatic gain control (AGC) target of 3E6 and a maximum injection time of 50 milliseconds. Precursor peptide ion isolation width for MS2 fragment scans was 1.4 m/z, and the top 12 most intense ions were sequenced. All MS2 spectra were acquired at a resolution of 15,000 with higher energy collision dissociation (HCD) at 27% normalized collision energy. An AGC target of 1E5 and 100 milliseconds maximum injection time was used. Dynamic exclusion was set for 5 s with a mass tolerance of ±10 ppm. Rawfiles were searched against the Uniprot Human database UP000005640 downloaded 11/2/2020 using MaxQuant v.1.6.14.0. Cysteine carbamidomethylation was considered a fixed modification, while methionine oxidation and protein N-terminal acetylation were searched as variable modifications. All peptide and protein identifications were thresholded at a 1% false discovery rate (FDR). Statistical analysis was performed on log2 transformed iBAQ and LFQ intensities using^1^ the R package ‘limma’, which normalizes and performs a Bayesian linear model statistical analysis and Benjamini-Hochberg false discovery rate adjustment (Ritchie et al., 2015). From the resulting data, putative interactors were selected by filtering for adjusted *p*-value < 0.05 and log-fold change > 0 from the IP with Caper antibody compared to the control IP with Rabbit Serum.

### Gene ontology

GO term enrichment analysis and pathway annotation network analysis was performed using the ClueGO plugin for Cytoscape (Bindea et al., 2009). Genes were clustered using GO terms for biological process and molecular function at GO tree levels from 4 to 10. Enrichment was tested using a right-sided hypergeometric test, and *p* values were adjusted to control the false-discovery rate (FDR) using the Benjamini-Hochberg procedure (cutoff significance was 0.05).

### Negative gravitaxis behavioral assay

A candidate screen, using a subset of the identified Caper interacting proteins and RNAs was carried out to identify modifiers of *caper* adult negative geotactic behavior. Fifty-one genes encoding protein interactors and RNA targets of Caper were tested with the gravitaxis assay (Supplementary Table S2). Mutant lines for these potential modifiers were crossed to *caper ^−/−^* flies to generate transheterozygotes, and gravitaxis analysis was performed using transheterozygous animals. Mutant lines for *caper* and for each individual candidate gene were outcrossed to *yw* to serve as controls, and the climbing speeds of transheterozygous animals were compared to both *caper ^+/−^* and “candidate gene” ^+/−^ controls. Importantly, we chose to screen transheterozygotes since lowering the dosage of candidate genes in a *caper ^−/−^* background generally results in lethality. The gravitaxis assay was performed on 10-day old flies grouped in 8–10 flies per trial. Flies were kept at RT. Males and females were separated for the assay as previous data has shown that *caper* dysfunction exhibit a sex-bias in gravitaxis phenotypes (Olesnicky et al., 2017). For each trial, flies were transferred to a graduated cylinder without carbon dioxide anesthesia. After 30 sec of acclimation, the flies were tapped three times to the bottom. The time it took for 50% of the flies to climb between two points of the cylinder was recorded. At least 10 trials were conducted per genotype per sex. Vials were monitored for a maximum of 90 s. If half the flies had not climbed between the two points by this time, the climbing time was recorded as “90 s” and treated as right censored data in the statistical analysis. Since data were censored, climbing speeds were analyzed using a parametric survival model from the R package, ‘survival’ (Terry, 2023). To choose the best model, we used Akaike information criterion (AIC) to compare models assuming the following distributions: Weibull, gaussian, exponential, log-normal, logistic, and log-logistic. Log-logistic models consistently had the lowest AIC values and were thus used for analyzes. The full factorial model included genotype, sex, and a genotype by sex interaction. Sex was excluded from the model in a few cases because only one sex was viable. Anova tables (type II sum of squares) were obtained using the R package ‘car’ (Fox and Weisberg, 2019). If a significant interaction or genotype effect was observed, *post hoc* tests were performed with the ‘emmeans’ package using Tukey’s correction for multiple testing (Lenth et al., 2021). Candidate genes were classified as modifiers when transheterozygotes differed in climbing speed from both controls.

## Results

### Identification of Caper interacting proteins and RNA targets in *Drosophila* heads

**Figure 1:**
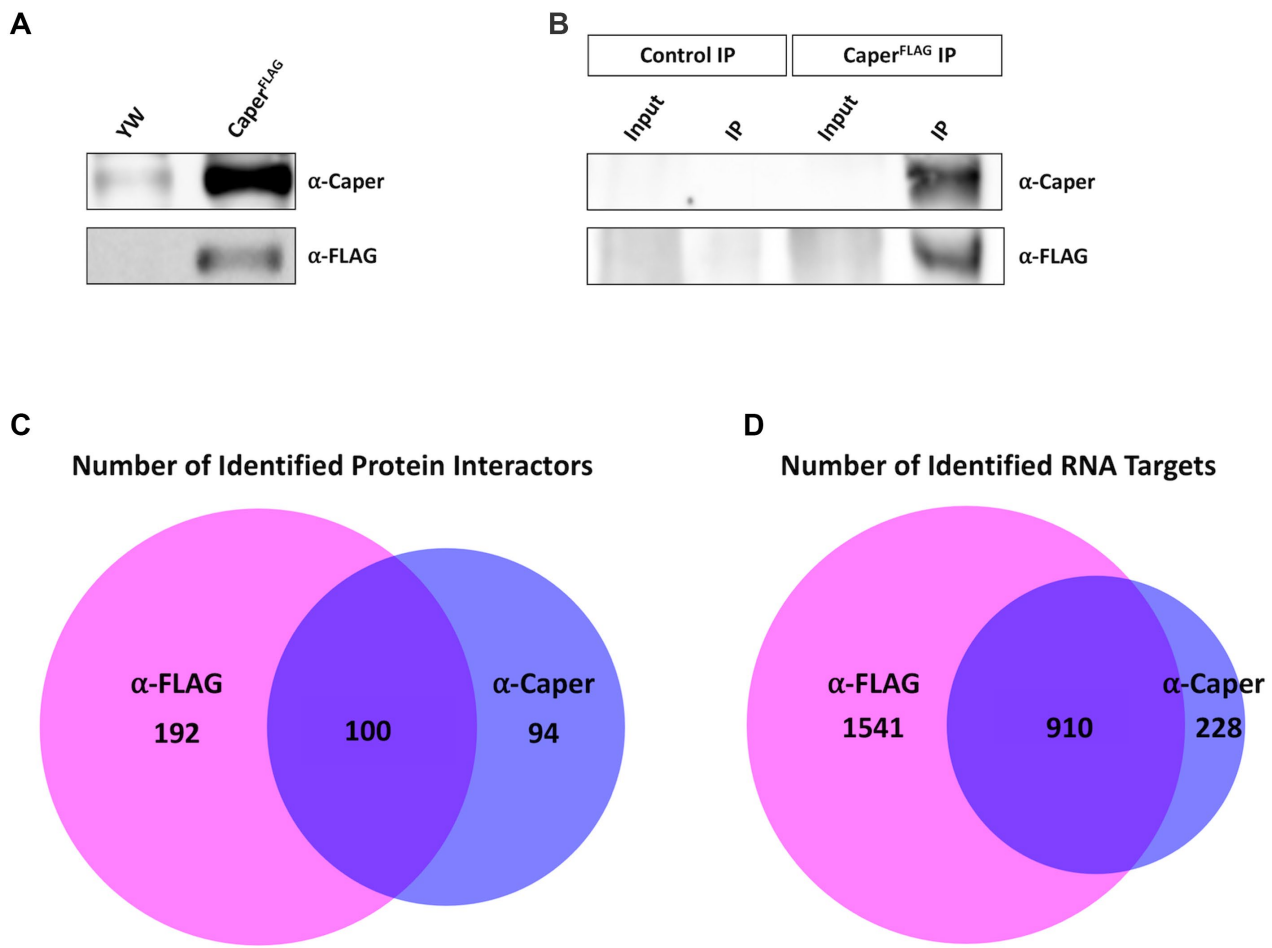
Immunoprecipitation of Caper utilized to identify interacting proteins and RNA targets. **(A)** Immunoblotting experiment demonstrates that Caper can be detected in *yw* and *caper^FLAG^* overexpression lines using an antibody specific to Caper. FLAG-tagged Caper is also detected in the *caper^FLAG^* overexpression lines. **(B)** Immunoblotting of immunoprecipitation experiments demonstrates Caper was successfully pulled down using both a Caper antibody and a FLAG antibody in *caper^FLAG^* overexpression lines. **(C)** Caper interacting proteins from the immunoprecipitation were identified using LC–MS. 292 proteins were identified with the FLAG antibody, 194 proteins were identified with the Caper antibody, and 100 proteins overlapped from both immunoprecipitations. **(D)** RNA targets of Caper were identified using RNA immunoprecipitation sequencing (RIPSeq). 2,451 RNA targets were identified with the FLAG antibody, 1,138 RNA targets were identified with Caper antibody, and 910 RNA targets overlapped from both immunoprecipitations.

To identify Caper protein interactors and RNA targets, Caper was immunoprecipitated from adult *Drosophila* heads, to enrich for neural tissue, and samples were analyzed using RNA sequencing and mass spectrometry. To this end, FLAG-tagged Caper was overexpressed in flies utilizing the *Gal4-UAS* system driven by a heat shock promoter. Immunoprecipitation was performed with two different antibodies, a polyclonal antibody specific to Caper (Titus et al., 2021) and a commercially available monoclonal antibody specific to the FLAG tag. We chose to use two different antibodies to provide an independent verification of Caper interacting proteins and target RNAs. Both antibodies were tested for efficacy and specificity by performing Western blotting analysis using lysates derived from adult *Drosophila* heads from the *yw* control line and the *UAScaperFLAG* overexpression line. The FLAG antibody detected a band of approximately 80 kDa in lysates derived from the *UAScaperFLAG* overexpression line that was not observed in lysates derived from *yw* control heads, as previously described (Titus et al., 2021; Figure 1A). Additionally, the Caper-specific polyclonal antibody detected a band at the same position in lysates derived from both *yw* and *UAScaperFLAG* overexpression lines, but at higher levels in heads overexpressing *caper* (Figure 1A). Immunoblotting of immunoprecipitated samples for both beads conjugated with anti-FLAG antibodies and beads conjugated with anti-Caper antibodies successfully detected Caper in both lysates, whereas Caper was not detected in the respective control immunoprecipitations conjugated only with Rabbit serum (Figure 1B). We note that Caper is not detected in the input lanes of the resulting Western blots because the high concentration of Caper in the immunoprecipitation samples (IPs) overshadows the input samples.

Protein interactors were identified through liquid chromatography-mass spectrometry (LC–MS) of the IPs and RNA targets were identified through RNA sequencing of RNA isolated from the IPs. In combination, 386 proteins were identified as potential interactors of Caper between the FLAG IP and Caper IP, with 192 proteins unique to the FLAG IP, 94 proteins unique to the Caper IP, and 100 proteins overlapping in both IPs (Figure 1C; Supplementary Table S3). Furthermore, a total of 2,679 RNA targets were identified through RNA-sequencing with 1,541 targets unique to the FLAG IP, 228 targets unique to the Caper IP, and 910 RNA targets overlapping in both IPs (Figure 1D; Supplementary Table S4). Twelve putative RNA targets were independently verified from the FLAG IP sequencing results using RT-PCR. To this end, RNA was isolated from three separate biological replicates of anti-FLAG immunoprecipitates derived from adult brain tissue of the *UAScaperFLAG* overexpression line, as well as their respective inputs. As a control for nonspecific binding, in three separate biological replicates brain lysates derived from *yw* control flies were incubated with anti-FLAG beads. The following 12 targets are enriched in the FLAG IPs compared to their mock IP controls: *qkr58E-1*, *chic*, *unc-115a*, *pros*, *lola, zfh1*, *acn*, *unc-115b*, dlg1, *tutl*, *brp*, and *chinmo* (Supplementary Figure S1).This further supports the validity of the RIPseq data set. The increased pull down of protein interactors and RNA targets in the FLAG IPs is likely due to a higher efficacy when utilizing commercial beads directly conjugated with FLAG antibody compared to beads incubated with the polyclonal anti-Caper antibody. However, the high degree of overlap between the two IPs in protein interactors and RNA targets suggests that these represent *bona fide* Caper target RNAs and protein interactors. We do note that one caveat to our approach of overexpressing Caper is that we are unable to differentiate between endogenous Caper interactors and those resulting from overexpression of Caper. However, due to the fact that a high number of interacting proteins are conserved interactors of human RBM39 suggests that our approach still identifies relevant interactors (see below).

### GO term analysis on interacting proteins of caper

**Figure 2:**
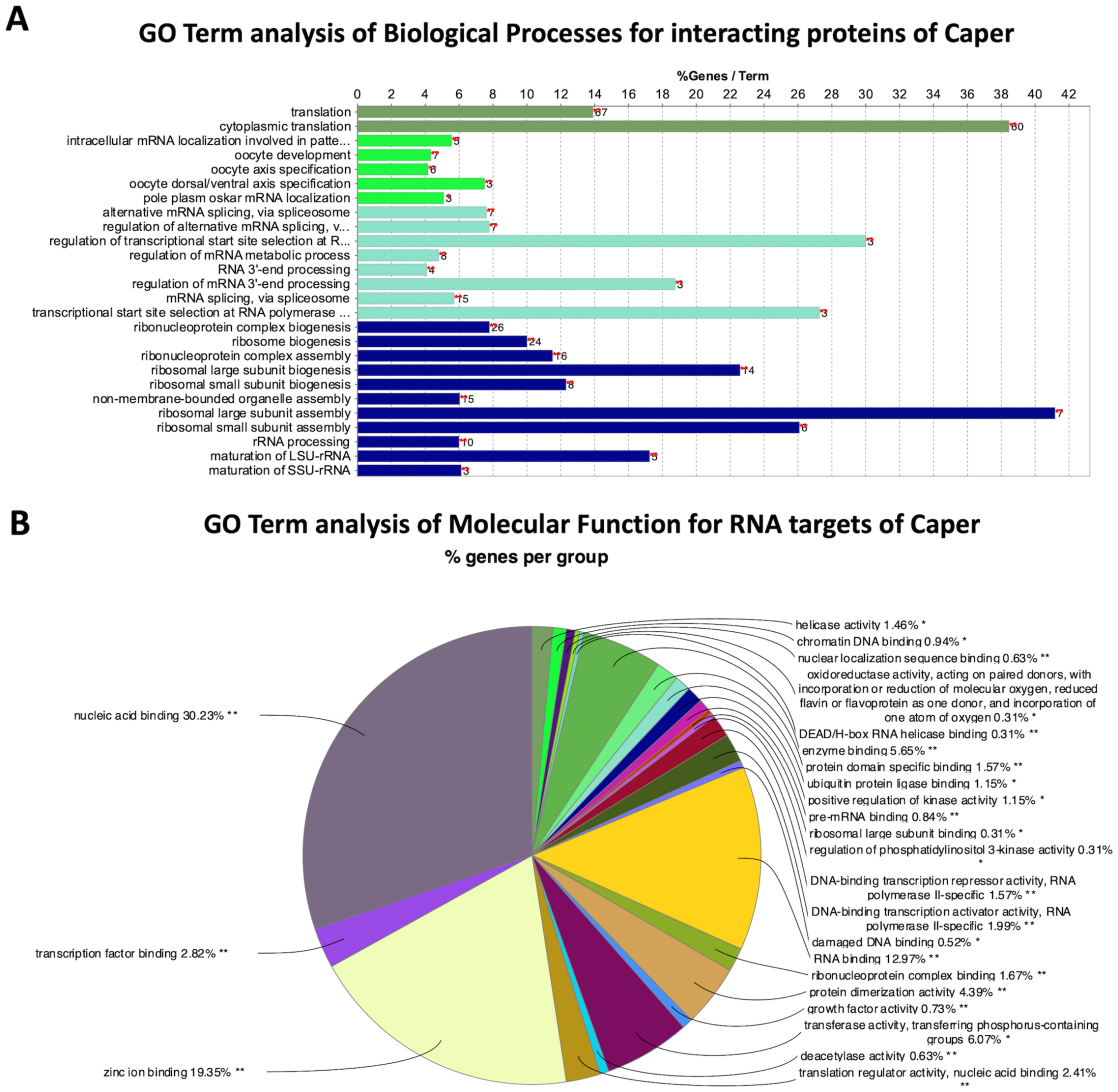
GO Term analysis reveals genetic pathways for Caper interactors and RNA targets. **(A)** Bar chart representing the GO terms of biological processes enriched by protein interactors of Caper by the percent of genes found for each term. The number to the right of each bar represents the number of genes found for that term. **(B)** Pie chart representing the group of GO terms of molecular functions enriched by the RNA targets of Caper. Each pie segment is a representation of the percent of genes found for each group.

GO Term analysis of biological processes was performed on the 100 overlapping proteins from the FLAG and Caper IPs utilizing the GO term analysis tool, ClueGO (Bindea et al., 2009). The GO Term analysis resulted in the enrichment of 34 GO Terms (Figure 2A; Supplementary Table S5). Interestingly, among the most highly enriched GO Terms are ribosomal assembly and cytoplasmic translation (Figure 2A). Specifically, several cytoplasmic ribosomal subunits, eukaryotic translation initiation factor, and poly-A binding protein coimmunoprecipitated with Caper. This data suggests that Caper may play a novel role in translational regulation, which is corroborated by the fact that Caper is detected in the cytoplasm of *Drosophila* larval neurons and interacts with the translational regulator Fragile X Messenger Ribonucleoprotein (FMRP) (Titus et al., 2021).

Additionally, Caper interacting proteins were enriched for the GO term “regulation of alternative mRNA splicing” (Figure 2A), which aligns with studies that have previously identified a role for *Drosophila* Caper and its human and mouse ortholog RBM39 in alternative splicing (Park et al., 2004; Dowhan et al., 2005; Huang et al., 2012; Ashton-Beaucage et al., 2014; Brooks et al., 2015; Mai et al., 2016; Stepanyuk et al., 2016; Stegeman et al., 2018). Specifically, several interactors of Caper that have been implicated in mRNA splicing include B52 (Liu and Bossing, 2016; Zhang et al., 2018; Srivastava et al., 2021), Acinus (Acn) (Rodor et al., 2016), and Splicing regulatory protein 54 (Srp54) (Garcia-Garcia et al., 2017). Finally, Caper also coimmunoprecipitated with U2 small nuclear riboprotein auxiliary factor 50 (U2af50), one of the subunits of U2 auxiliary factor (U2af), which is critical for the recruitment of the U2 small nuclear ribonucleoprotein (snRNP) to the 3′ splice site to initiate spliceosomal assembly (Zamore and Green, 1989). This data verifies and supports the role of *caper* in alternative splicing and provides validation for the efficacy of the IPs.

Interestingly, Caper interacting proteins were also enriched for the GO Term “oocyte development.” This is not necessarily surprising as there is a significant overlap of genes, particularly RBP-encoding genes, that are expressed and function in the germline and within neurons (Olesnicky et al., 2014). Among the interacting proteins enriched for the oocyte development GO Term are Syncrip (Syp), and the subunits for casein kinase II (CKII): casein kinase IIα (CkIIα), and casein kinase IIβ (CkIIβ). *syncrip* is important for synaptic plasticity of neuromuscular junctions in *Drosophila* larvae and in the determination of neuroblast fate (Halstead et al., 2014; McDermott et al., 2014; Yang et al., 2017; Rossi and Desplan, 2020). *syncrip* is also critical for axis specification during germline development (McDermott et al., 2012). Furthermore, *CKII* is important for ribosome biogenesis, cell growth in neuroblasts (Hovhanyan et al., 2014) and for lipid metabolism during oogenesis (McMillan et al., 2018). Thus, Caper interacts with proteins that play a role in both neurodevelopment and oogenesis and points to a possible role for *caper* within the germline.

### Caper targets its own RNA

**Figure 3:**
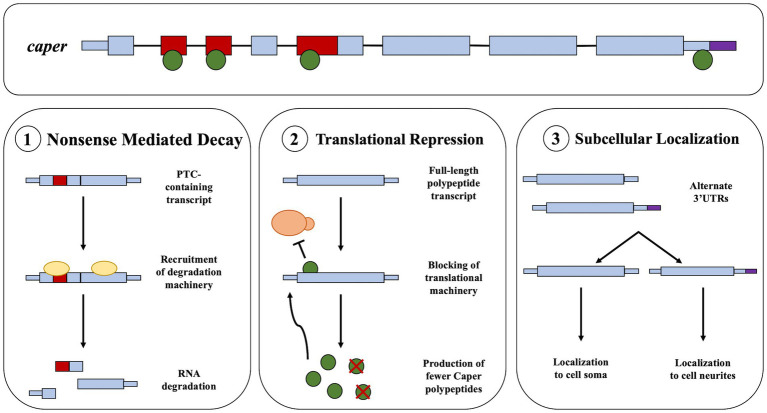
Potential models for the autoregulation of Caper. This figure presents a pre-mRNA model of *caper* including exons and introns. The green circles represent the Caper protein. **(1)** The first model suggests that the binding of Caper to its own mRNA could result in the increased inclusion of a PTC that results a negative feedback loop through the process of nonsense mediated decay. **(2)** The second model suggests that Caper may bind its own mRNA resulting in a negative feedback loop through translational repression. **(3)** The third model suggests that Caper binds to its own mRNA to result in the selection of an alternative 3’UTR which could impact the subcellular localization of *caper* mRNA.

We find that Caper binds to its own mRNA. It is not unusual for RBPs and alternative splicing factors to engage in auto-regulation of their own mRNAs (Dredge et al., 2005; Buratti and Baralle, 2012; Humphrey et al., 2019; Müller-Mcnicoll et al., 2019). It should be noted that *caper* has seven different spliceforms, five of which contain poison exons, exons that contain premature termination codons (PTCs) that either result in nonsense mediated decay or the translation of a truncated protein. Furthermore, manual investigation of sequencing read counts reveals that PTC-containing exons of *caper* mRNA have higher reads in the immunoprecipitation samples than input samples, suggesting Caper might specifically regulate its own mRNA by regulating poison exon inclusion. Therefore, we suggest three different models by which Caper regulates its own mRNA. The first is that Caper regulates the splicing of its RNA to select for the PTC-containing exons as a negative feedback loop (Figure 3). Second, based on the potential role that Caper plays in translational regulation, Caper may act as a translational regulator of its own RNA (Figure 3). Finally, *caper* has two spliceforms that create identical full-length polypeptides with differing 3’UTRs. Thus, Caper may alter the splicing of the 3’UTR region to regulate subcellular localization, specifically regulating the localization of *caper* transcripts to the soma or neurites in neurons (Figure 3). This is supported by the fact that 3’UTR length has been determined as a deciding factor in the localization of transcripts to neurites in cell culture (Taliaferro et al., 2016). It is important to note that none of these hypotheses are mutually exclusive. Indeed, RBPs are well known to play myriad roles in RNA regulation.

### Caper RNA targets include those encoding several of its protein interactors

When the complete set of 386 interacting proteins pulled down in either the anti-Caper or anti-Flag IPs is compared to the complete set of targets pull-downed in either RNA immunoprecipitation experiment, 103 of the proteins that Caper interacts with were also identified as RNA targets of Caper (Supplementary Table S6). Previous research has already highlighted that RBPs, and specifically splicing factors regularly engage in cross-regulation, where two interactors will regulate the RNA processing of one another (Fu and Ares, 2014; Brooks et al., 2015).

**Figure 4:**
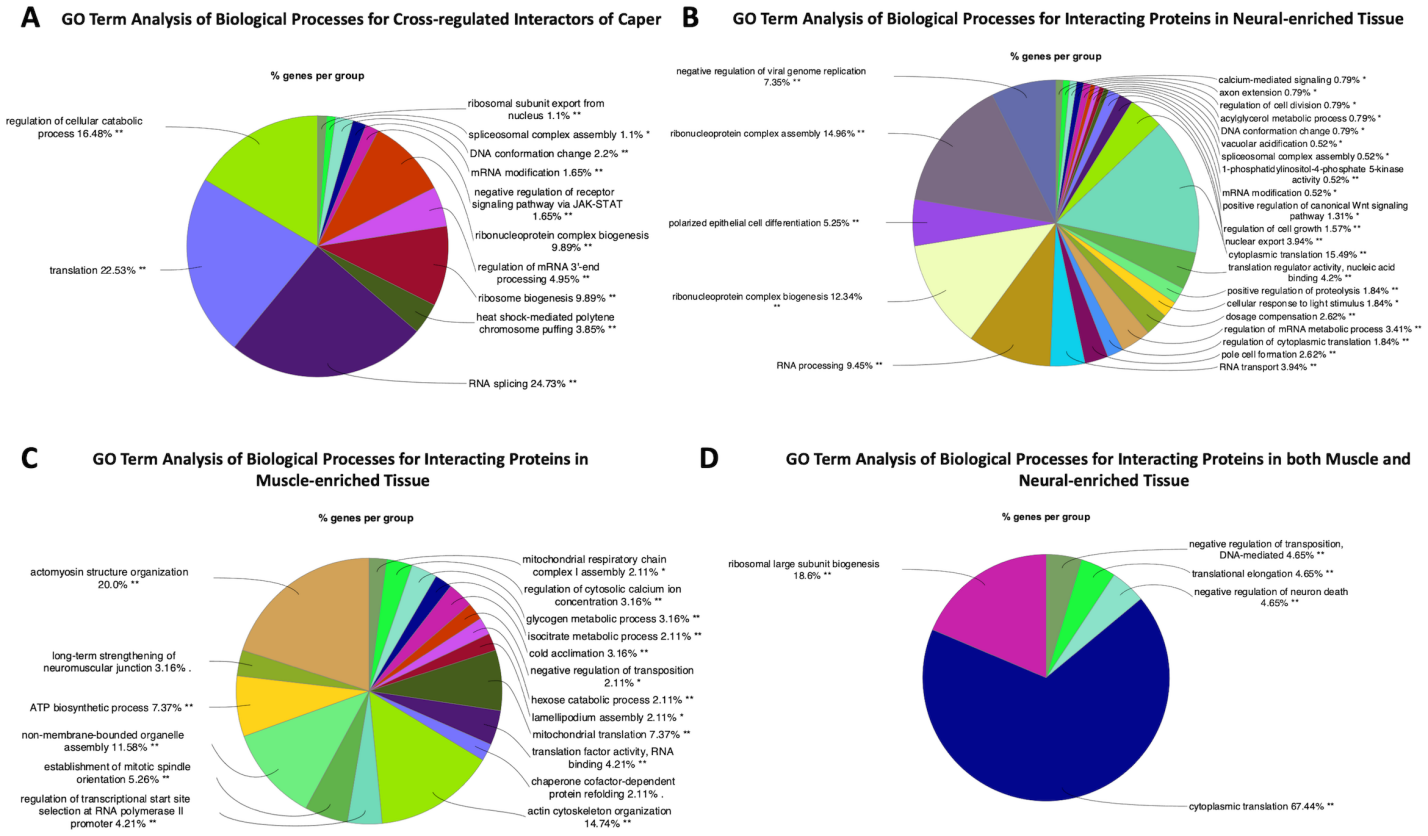
GO Term analysis reveals differences and similarities between Caper interactors in neural tissue compared to muscle tissue. This figure presents GO Term analysis for **(A)** interacting proteins that are also RNA targets of Caper, **(B)** GO Term analysis for interacting proteins that were pulled down exclusively in neural-enriched tissue, **(C)** GO Term analysis for interacting proteins that were pulled down exclusively in muscle-enriched tissue, and **(D)** GO Term analysis for interacting proteins that were pulled down in both neural and muscle-enriched tissue. Each pie segment is a representation of the percent of genes found for each group.

GO Term analysis of interacting proteins whose RNAs are also regulated by Caper includes many terms associated with alternative splicing and translation regulation. Other GO terms include the terms for mitotic cell cycle and pole cell formation (Figure 4A; Supplementary Table S6). However, closer inspection of the genes clustered in the latter two GO term categories indicate that these RNAs encode RBPs, suggesting that the effects are likely due to downstream interactions of targeted RNAs. Overall, these data suggest that *caper* likely engages in cross-regulatory mechanisms to regulate the mRNAs of many of its protein interactors.

### Comparison of protein interactors of Caper in nervous and muscle tissue

We performed separate immunoprecipitation experiments using the Caper antibody on dissected adult thorax muscle. Unfortunately, technical issues precluded our ability to also perform immunoprecipitations for RNA and protein interactors with the FLAG antibody in muscle tissue, or for RNA immunoprecipitation with the Caper antibody. Nonetheless, we identified Caper interacting proteins in muscle-enriched tissue and compared this to the protein interactors identified from neural-enriched tissue from *Drosophila* heads. To this end, of the 194 protein interactors identified in the neuron-enriched immunoprecipitation and the 140 protein interactors identified in the muscle-enriched immunoprecipitation, only 44 of the interacting proteins overlap in both tissue types (Supplementary Table S7). Interestingly, GO Term analysis of biological processes of interacting proteins pulled down in nervous tissue only was mostly enriched for terms associated with translation and splicing. However, it does include GO terms such as silencing via micro RNAs and signal transduction (Figure 4B; Supplementary Table S8). GO Term analysis of the biological processes of interacting proteins pulled down specifically in muscle tissue identified some unique GO Terms such as adult muscle development, protein folding, sarcomere organization, myofibril assembly, and mitochondrial translation (Figure 4C; Supplementary Table S8). This demonstrates that despite many overlapping functions, Caper may have distinct functions in different tissue types, as a result of differential interaction with proteins and potentially RNA targets.

In muscle-enriched tissue there is also an enrichment of mitochondrial and cytoskeletal-associated proteins pulled down compared to neural-enriched tissue. It is uncertain as to why Caper interacts with more mitochondrial associated proteins in muscle than in nervous tissue. However, this could be due to the potential of higher levels of mitochondria and mitochondrial DNA present in muscle tissue compared to neural tissue (D’Erchia et al., 2015; Herbers et al., 2019; Moreno-Loshuertos and Fernández-Silva, 2020). The association of Caper with the cytoskeleton is not surprising given the relationship between RBPs and the cytoskeleton. Many RNA-binding proteins and RNA molecules form ribonucleoprotein (RNP) granules to perform post-transcriptional regulation and to be transported to various subcellular regions (Barbee et al., 2006; Kato and Nakamura, 2012; Hubstenberger et al., 2017; Christou-Kent et al., 2020). Furthermore, the cytoskeleton has been demonstrated to be crucial in both the formation and transport of RNP granules (Mamon et al., 2017; Chudinova and Nadezhdina, 2018). Finally, it is important to note that several interactors from both neural and muscle-enriched tissue were associated with NMJ development. This aligns well with the aberrant NMJ morphology previously observed with *caper* dysfunction both ubiquitously and in motoneuron specific knockdown of *caper*. However, muscle specific knockdown of *caper* results in distinct and less severe NMJ phenotypes as compared to motoneuron and glia specific knockdown of *caper* (Titus et al., 2021). Taken together, these results suggest that *caper* functions in the muscle, to some extent, to regulate NMJ development.

GO Term analysis of the biological processes of interacting proteins pulled down in both muscle and nervous tissue demonstrates that most of the interactors of Caper across tissue types are involved in translation and splicing (Figure 4D). Twenty-seven of the 44 overlapping proteins were identified as being ribosomal sub-units, further implicating Caper in translational regulation. By contrast, 6 of the 44 overlapping proteins were identified as being associated with alternative splicing, which suggests that Caper may play a significantly larger role in translational regulation than previously expected.

### Conserved interactions observed in humans

Using data from BioGrid we were able to identify 130 putative protein interactors from our IP experiments that had orthologs in humans that physically interacted with RBM39, one of two human orthologs to *caper* (Stark et al., 2006; Supplementary Table S9). Of these 130 interactors that are conserved, 51 interactors were pulled down in the IP performed on heads, 46 interactors were pulled down in muscle, and 33 were pulled down in both heads and muscle. GO Term analysis for Caper interactions that are potentially conserved in humans reveals 42 GO Terms for the Caper interactors and 115 GO Terms for the RBM39 interactors (Supplementary Tables S10, S11). This suggests that RBM39 could have more diverse functions in humans than *caper* has in *Drosophila.* However, there are several overlapping GO terms including various terms associated with translation, ribosomal assembly, and RNA processing. This suggests that many of the core functions of *caper* are conserved in humans. Furthermore, 3 of the conserved interactors are identified as modifiers of adult gravitaxis: *acinus (Acn), syncrip (syp), and purine-rich binding protein-alpha* (pur-alpha). Finally, FMRP, which was identified as an interactor of Caper in previous publications is also identified as a conserved interactor in humans (Titus et al., 2021). Overall, this data demonstrates that many interactions are likely conserved in humans and serves to further validate the results of the IP experiments.

### Candidate interactors modify the *caper* gravitaxis phenotype

To independently verify the results of these co-immunoprecipitation experiments and to begin to elucidate to which phenotypes these protein and RNA interactors are relevant, we performed a candidate-based modifier screen on a subset of the identified Caper interacting proteins and RNA targets. To this end, we chose to identify genes that modify the *caper* mutant adult gravitaxis phenotype. We have previously shown that *caper* dysfunction results in slower climbing speeds in the well-established gravitaxis pathway (Olesnicky et al., 2017; Titus et al., 2021). Furthermore, we have also shown that FMRP, which co-immunoprecipitates with Caper in neuronal tissue, modifies gravitaxis behavior of *caper* mutants, lending credence to this strategy. We considered genes to be modifiers when transheterozygotes carrying a mutant allele at each locus had phenotypes that differed from both heterozygous controls.

**Figure 5:**
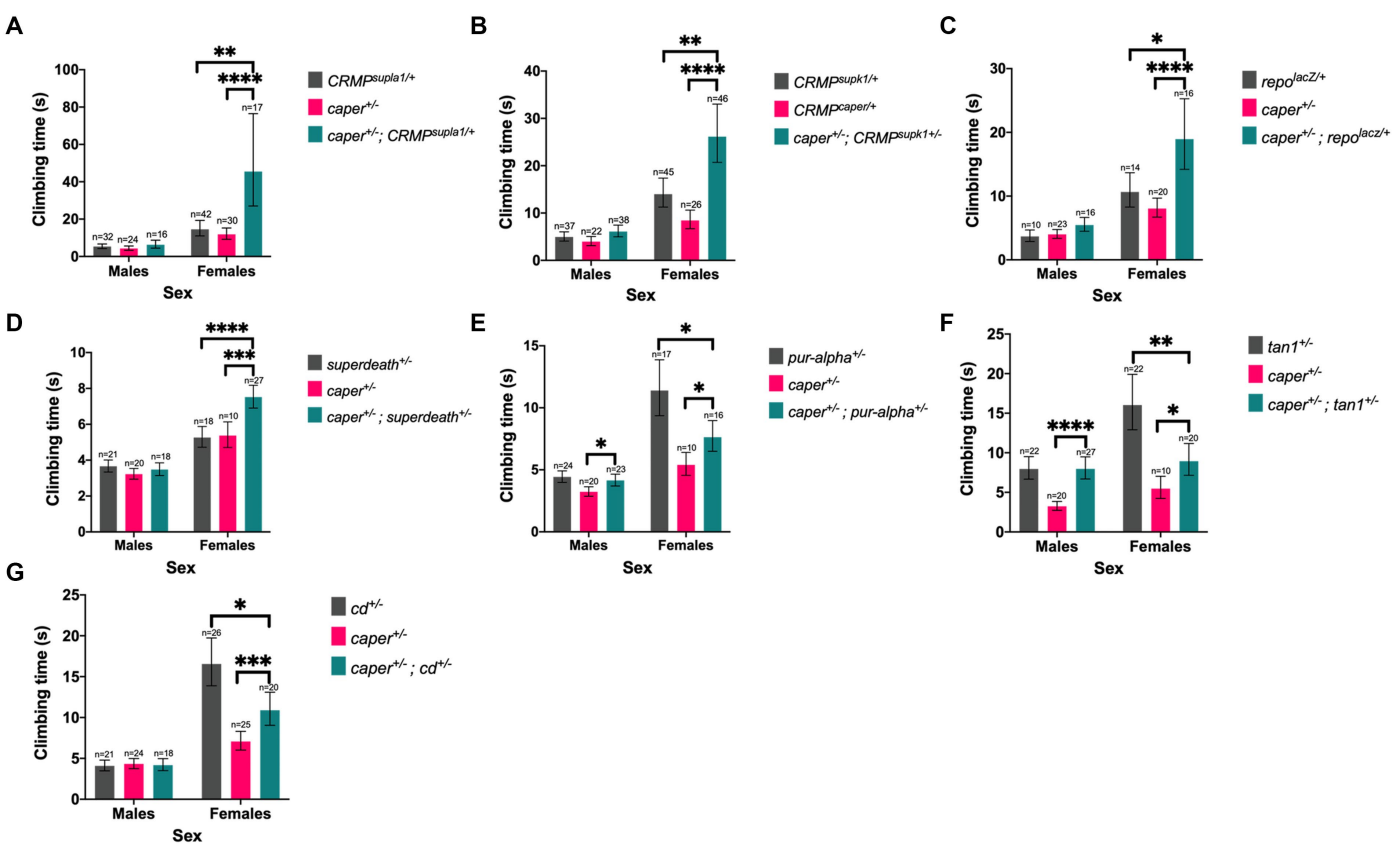
Six candidate genes modify the Caper gravitaxis phenotype in females. Mutations in *CRMP* **(A,B)**, *repo* **(C)**, *superdeath* **(D)**, *pur-alpha* **(E)**, *tan* **(F)**, and *cd* **(G)** modify gravitaxis defects in *caper* mutant females, but males are unaffected (genotype x sex interaction). Bars represent estimated marginal mean climbing times from log-logistic survival models and error bars represent 95% confidence intervals. *p ≤ 0.05; **p ≤ 0.01, ***p ≤ 0.001, ****p ≤ 0.0001.

**Figure 6:**
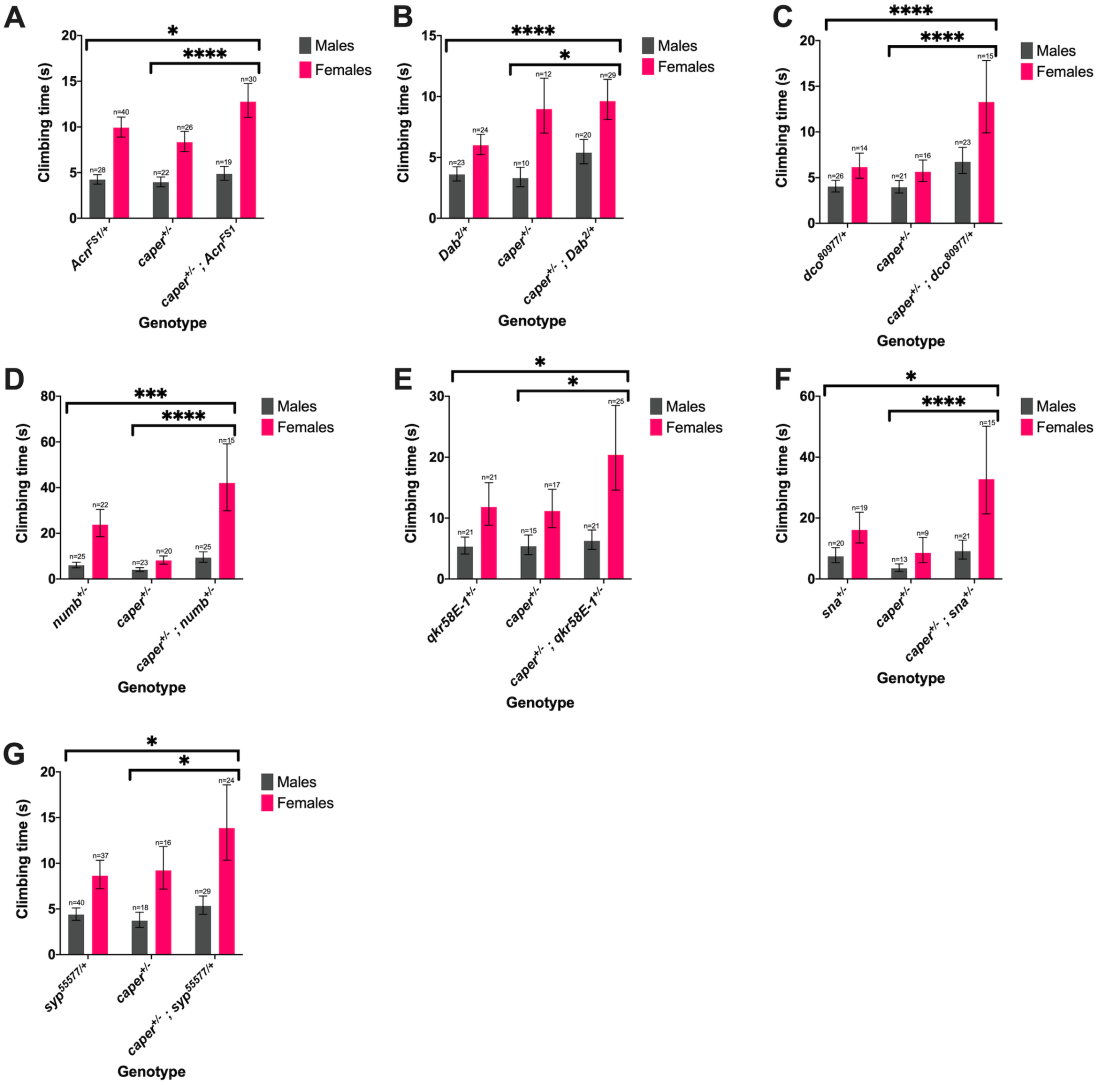
Seven candidate genes modify the Caper gravitaxis phenotype in both sexes. Mutations in *Acn* **(A)**, *Dab* **(B)**, *dco* **(C)**, *numb* **(D)**, *qkr58E-1* **(E)**, *sna* **(F)**, *syp* **(G)** modify gravitaxis defects in both caper mutant males and females. Bars represent estimated marginal mean climbing times from log-logistic survival models and error bars represent 95% confidence intervals. *p<=0.05; **p<=0.01, ***p<=0.001, ****p<=0.0001.

Thirteen candidate genes were found to modify the Caper gravitaxis phenotype: *numb, Acn, pur-alpha, snail (sna), cardinal (cd), suppressor of ER stress-induced death (superdeath), Collapsin Response Mediator Protein (CRMP)* (both mutant lines tested)*, disabled (dab)* (one of two mutant lines tested)*, reversed polarity (repo)* (one of two mutant lines tested), *discs overgrown (dco)* (one of two mutant lines tested), *quaking related 58E-1 (qkr58E-1)*, *tan (t)* (one of two mutant lines tested), and *syp* (one of four mutant lines tested). Six of these genes had stronger effects in females than males, as revealed by significant genotype x sex interactions (Figure 5; Supplementary Table S12). For the remaining seven genes, both sexes were equally affected, as indicated by a significant genotype effect without a significant genotype x sex interaction (Figure 6; Supplementary Table S13). We note that although there was a significant interaction for *numb*, female transheterozygotes only differed from one of the controls, so we instead report the overall genotype effect. In 10/13 cases, transheterozygotes displayed reduced climbing speed compared to both controls (Figures 5A–D, 6). While our experimental design does not directly test whether these effects are non-additive and thus indicative of a genetic interaction, we note that the severity of the climbing deficit in transheterozygotes in several cases strongly suggests that effects are non-additive. For three candidate genes (Figures 5E–G), transheterozygotes had an intermediate phenotype between the controls, which suggests an antagonistic genetic interaction with *caper*. Altogether, the results of the modifier screen provide additional independent support for co-immunoprecipitation experiments that identify direct or indirect interactors of Caper.

## Discussion

### Caper as a regulator of post-transcriptional regulation via RNP granules

In order to better understand the specific post-transcriptional functions of Caper in neuronal cells, we identified a network of proteins and RNAs that interact with the RBP Caper. One of the primary methods of post-transcriptional regulation is through the formation of ribo-nucleoprotein (RNPs) granules that include the association of RNAs and RNA-binding proteins through liquid–liquid phase separation (Barbee et al., 2006; Kato and Nakamura, 2012; Hubstenberger et al., 2017; Christou-Kent et al., 2020). Some of the most commonly identified RNP granules include nuclear granules such as paraspeckles, Polycomb, and Cajal bodies; as well as cytoplasmic granules including processing bodies (P-bodies), stress granules, and Staufen-containing granules (Barbee et al., 2006; Hubstenberger et al., 2017). It has been demonstrated that mRNAs may be targeted to P-bodies to undergo translational repression, miRNA gene-silencing, nonsense-mediated decay pathways, and storage for later activation (Brengues et al., 2005; Bhattacharyya et al., 2006; Decker and Parker, 2012; Ross Buchan, 2014).

P-body protein components have been identified in HEK293 human epithelial cell lines, and importantly several are orthologous to proteins identified as interactors of Caper including DEAD box helicase 6 (DDX6) / maternal expression at 31B (Me31b), Insulin Like Growth Factor 2 mRNA binding protein 2 (IGF2BP2) /Imp, Argonaute RISC component 1 and 2 (AGO1 and AGO2) /Argonaute 2 (AGO2), and UPF1 RNA Helicase and ATPase (UPF1) /Upf1 RNA helicase (Upf1). Since many Caper interacting proteins are known granule components, Caper may be a component of P-bodies and Staufen-containing granules. Furthermore, all of these RBPs are recognized as being involved in translational repression, mRNA decay, or the miRNA pathway (Hubstenberger et al., 2017). This supports the possibility that Caper plays a role in these additional RNA regulatory pathways. This is further supported by the GO term analysis for Caper interacting proteins that shows enrichment of the GO terms for translation, mRNA processing, and production of siRNA involved in post-transcriptional gene silencing by RNA.

Furthermore, several components of the coding region determinant (CRD) mediated complex were also identified in P-bodies from HEK293 cells and the fly orthologous proteins co- immunoprecipitate with Caper. These proteins include Heterogenous Nuclear Ribonucleoprotein U/CG30122, Synaptotagmin Binding Cytoplasmic RNA Interacting Protein (SYNCRIP)/Syp, Insulin Like Growth Factor 2 mRNA binding protein 1 (IGF2BP1)/Imp, and DExH-box Helicase 9 (DHX9)/maleless (mle) (Hubstenberger et al., 2017). These components were also demonstrated to promote the stability of the myc mRNA through the interaction of the CRD sequence found in the 3’UTR (Weidensdorfer et al., 2009). Interestingly, *myc* mRNA was pulled down with Caper in the FLAG IP suggesting that *myc* is also an RNA target of Caper, and that Caper may also function to promote the stability of *myc* and other mRNA targets.

It is also likely that Caper associates with Staufen-containing RNP granules, which have been demonstrated to mediate translational repression and mRNA transportation (Barbee et al., 2006; Kiebler and Bassell, 2006). Although Caper does not interact with Staufen (Stau) directly in our experimental conditions, it does interact with several other proteins that have been identified as components of the Staufen-containing transport RNPs (Barbee et al., 2006; Kiebler and Bassell, 2006). These proteins include Fmrp, Barentz (Btz), Me31b, Ypsilon schachtel (Yps), Imp, AGO2, and Upf1 (Barbee et al., 2006). Furthermore, the majority of these proteins have also been demonstrated to be components of maternal RNP granules and P-body granules, further supporting Caper’s interaction with P-bodies and highlighting a potential interaction with maternal RNP granules (Barbee et al., 2006). The latter of which is supported by the enrichment of the GO term oocyte development for Caper interacting proteins. Finally, given the identified role of Staufen-containing granules in RNA localization, this may suggest that Caper plays a role in subcellular RNA localization. This is consistent with the enrichment of GO Terms such as intracellular mRNA localization involved in pattern specification process and pole plasm oskar mRNA localization (Barbee et al., 2006; Kiebler and Bassell, 2006). Taken together, the identification of Caper protein interactors underscores the association of Caper with many cytoplasmic RBPs and provides additional support for Caper having myriad roles in post-transcriptional gene regulation beyond alternative splicing.

### Caper protein interactors have roles in neurodevelopment

Despite the established role of *caper* in the regulation of the development of several neural subtypes (Olesnicky et al., 2017; Titus et al., 2021), there was not an enrichment of gene ontologies associated with neuron development for Caper protein interactors. Nonetheless, we previously uncovered a direct interaction between Caper and the RNA-binding protein Fmrp through co-immunoprecipitation and co-localization. Furthermore, *caper* and *Fmr1* demonstrated a genetic interaction in pathways regulating adult gravitaxis behavior (Titus et al., 2021). Therefore, it is not surprising that several of the interacting proteins pulled down with Caper have been demonstrated to regulate neural development and maintenance, including B52, IGF-II mRNA binding protein (Imp) and Syp. Depletion of *B52* results in an increase in the axon length of dMP2 neurons in *Drosophila* embryos and differential splicing of *Choline-acetyltransferase* (*ChAT*) which causes reduced production of the neurotransmitter acetylcholine (Liu and Bossing, 2016). The RBPs Imp and Syp act as temporal cues that have opposing roles in determining neuroblast fate (Yang et al., 2017; Rossi and Desplan, 2020). *Imp* dysfunction has also been demonstrated to impact the axon growth of γ neurons of the mushroom body in adult flies and the dendritic branching of class IV dendritic arborization (da) neurons in *Drosophila* larvae (Hattori et al., 2013; Medioni et al., 2014). Interestingly, dysfunction of *caper* has also been demonstrated to impact the dendritic branching of class IV da neurons, which suggests that *Imp* and *caper* may interact to regulate dendrite formation (Olesnicky et al., 2017). *Syp* has also been demonstrated as being critical for the synaptic plasticity and development of neuromuscular junctions in *Drosophila* larvae (Halstead et al., 2014; McDermott et al., 2014; Titlow et al., 2020). Since *caper* has also been demonstrated to be important in the development of larval neuromuscular junctions, *caper* and *Syp* may work coordinately to regulate NMJ morphogenesis (Titus et al., 2021).

### RNA targets of Caper function in neurogenesis, apoptosis and immune response

A GO Term analysis of the 910 overlapping target RNAs pulled down with both the Flag IP and Caper IP reveals that *caper* may regulate a broad range of biological processes, as highlighted by the identification of 507 GO Terms (Supplementary Table S4). Importantly, among the many enriched GO terms are several neurodevelopmental pathways including central nervous system development, photoreceptor cell fate determination, peripheral nervous system development, dendrite morphogenesis, ventral nerve cord development, neuroblast proliferation, and regulation of gliogenesis. This is in alignment with previous work that identified *caper* function as being important for the development of several neural subtypes, as well as, its role in visual function in the aging fly eye (Olesnicky et al., 2017; Stegeman et al., 2018; Titus et al., 2021). In particular, *caper* functions in multiple neural subtypes of the peripheral nervous system to direct dendrite morphogenesis of Class IV dendrite arborization neurons, axonogenesis of the neuromuscular junction, and in the development of proprioceptive neurons termed chordotonal neurons. Furthermore, knock down of *caper* function specifically within glia results in the strongest adult locomotor phenotypes, as compared to its knockdown pan-neuronally or within motor neurons (Olesnicky et al., 2017; Stegeman et al., 2018; Titus et al., 2021). Thus, the enrichment of these neuronal specific GO terms from the target RNA dataset is in line with established roles of Caper in neurogenesis.

Interestingly, GO term analysis of RNA targets also suggests that *caper* may play a role in immune response. GO terms indicating a role for *caper* in immune response include innate immunity, Toll signaling pathway, regulation of antimicrobial peptide production, regulation of antimicrobial humoral response, regulation of antifungal peptide production, and defense response to virus. Although neither *caper* nor its orthologs have been shown to play direct roles in the immune system, dysfunction of the immune system has been associated with neurodegenerative disease (Shrestha et al., 2014; Hammond et al., 2019; Dhankhar et al., 2020). Aberrant function of *caper* results in declining performance in gravitaxis assays that is exacerbated with age, which may be an indicator of neurodegeneration. Furthermore, knock down of *caper* or dysfunction of *caper* through a genetic lesion results in shortened lifespans of *Drosophila* adult animals compared to controls (Titus et al., 2021). This may suggest that *caper* has an immunological function that results in neurodegeneration when *caper* function is reduced. This hypothesis is supported by the fact that many immune pathways, including Toll signaling, are implicated in necroptosis of neurons (Andreone et al., 2020). Alternatively, *caper* may simply play a direct and distinct pleiotropic role in immune response.

Consistent with the possibility of neurodegeneration in *caper* mutant animals, among the enriched GO terms are positive regulation of cell death, apoptotic signaling pathway and programmed cell death involved in cell development, positive regulation of necrotic cell death, as well as, regulation of autophagy (Guo et al., 2018; Andreone et al., 2020). Indeed, neuronal cell death is a hallmark of neurodegeneration and aberrant autophagy has been implicated in the pathogenesis of various neurodegenerative diseases. Nonetheless, apoptosis is also a normal feature of neurogenesis, particularly during metamorphosis and neuronal remodeling. Given the ages of the fly heads used to generate neural enriched lysates for these analyses, it is also possible that *caper* regulates normal apoptosis and neuronal remodeling of the *Drosophila* brain (Yaniv and Schuldiner, 2016).

When analyzing GO terms for the molecular function of Caper RNA targets, 30 terms were enriched (Figure 2B; Supplementary Table S14), with the vast majority of them associated with either DNA or RNA-binding. This suggests that Caper may not directly impact many effector molecules, but instead regulates RNAs encoding transcription factors and RBPs resulting in a cascading impact on the expression of genes that impact neural development.

### Caper as a master regulator

We propose that *caper* be classified as a master regulator. First, a significant percentage of *caper* targets fall under the gene ontology molecular functions for nucleic acid binding, transcription factor binding, zinc ion-binding, translational regulator activity, and RNA binding. Thus, many Caper target RNAs are classified as RBPs and transcription factors. This suggests that *caper* rarely engages directly with effector molecules, but instead is part of hierarchical regulatory processes that result in indirect effects downstream of its targets. In other words, Caper regulates the initial steps of various signaling and regulatory pathways. Second, the gene ontology analysis of target RNAs for “biological processes” includes 507 terms varying from neurodevelopment, immune response, cytoskeletal organization, and gene silencing to name a few. This suggests that *caper* regulates many pathways, all which function in the proper development of the nervous system.

Other research also suggests that *caper* may be a master regulator. One study developed a large splicing network based off modENCODE data and RNAseq data. Here 10 network modules were identified, with each module defined as a set of nodes with “more dense connection patterns among their members than between their members.” Of these 10 modules, *caper* (*cg11266*) was identified as one of the top regulators for the module enriched in GO terms for organ development, locomotion, and neuron differentiation (Papasaikas et al., 2015). This is not surprising given that *caper* is important for the development of several neural subtypes, as well as, proper locomotion in larvae and adults (Olesnicky et al., 2017; Titus et al., 2021). Furthermore, the top regulators for every module contained at least one putative Caper RNA target based on the dataset presented here, suggesting Caper regulates other major regulators of gene expression. They also elucidated several central nodes within this splicing network. To this end, *caper* was identified as one of the 20 central-most network nodes. Finally, 11 of the other 20 central-most network nodes, where a node indicates a particular protein, are putative targets of *caper* (Papasaikas et al., 2015).

### Sex-specific regulation

We previously demonstrated that the dysfunction of *caper* impacts males more than females in several phenotypes including gravitaxis, grooming, lifespan, and neuromuscular junction morphology (Olesnicky et al., 2017; Titus et al., 2021). However, the mechanism for these sex-specific phenotypes is unclear. First, *caper* is an autosomally encoded locus, ruling out a simple X-linked effect. However, analysis of our overlapping RIP-seq datasets reveal that Caper may regulate the RNA of several genes involved in sex-determination through X-chromosome dosage compensation including *maleless* (*mle*), *male-specific lethal 1* (*msl-1*), *male-specific lethal 3* (*msl-3*)*, males absent on the first* (*mof*), *over compensating males* (*ocm*), and the *long non-coding RNA on the X 1* (*lncRNA:roX1*). Here we show that Caper also targets several RNAs in the *sex-lethal* (*sxl*) specific cascade that contributes to sexual development including *transformer* (*tra*), *transformer 2* (*tra2*), *fruitless* (*fru*), *doublesex* (*dsx*), and *sister-of-Sex-lethal* (*ssx*). Thus, Caper’s regulation of mRNAs associated with sexual development could explain the observed sex-bias in various neurological phenotypes.

*Sxl* utilizes the alternative splicing of genes such as *tra* and *msl-2* as part of an alternative splicing cascade that impacts sex determination and sexual differentiation (Penalva and Sánchez, 2003; Moschall et al., 2019). However, *Sxl* also undergoes splicing, which results in a full-length transcript found in females or a poison-exon containing transcript found in males (Penalva and Sánchez, 2003; Salz and Erickson, 2010). Expression of Sxl also results in a positive feedback loop in which the poison exon is excluded, inducing the production of more Sxl protein (Salz and Erickson, 2010). Interestingly, the paralog to *sxl, sister-of-sex-lethal* (*ssx*) inhibits the positive feedback loop of *sxl*, favoring the inclusion of the poison exon that results in the male variant of *sxl* (Moschall et al., 2019). Since *ssx* is a potential RNA target of caper, it is plausible that this creates a splicing cascade that impacts *sxl* and its downstream targets resulting in some of the sex-specific phenotypes we have observed previously (Olesnicky et al., 2017; Titus et al., 2021). Three additional mRNAs that *caper* targets that are involved in sexual determination include the RNA for the genes *fru*, *tra,* and *dsx*. All of these are part of a splicing cascade that begins with *sxl* (Penalva and Sánchez, 2003; Garner et al., 2018). Dysfunction of these genes can result in alteration of downstream selection for male or female spliceforms that can impact courtship behavior or morphology (Nilsson et al., 2000; Penalva and Sánchez, 2003). Furthermore, *fru*, *tra*, and *dsx*, have been implicated in the development of female-specific Insulin-like peptide 7-expressing (FS-Ilp7) oviduct motor neurons in females. The female-specific splicing of *fru and dsx* in males results in increased persistence of FS-Ilp7. However, the male-specific splicing of *tra* results in the loss of FS-Ilp7 in females (Garner et al., 2018). Thus, Caper may be an important regulator of the sex determination pathway. We should note, however, that global sex determination does not seem to be affected upon *caper* dysfunction, as genitalia of males and females forms normally (not shown). Thus, it is possible that Caper regulates only specific aspects of sex determination and development, for example, in a tissue specific manner.

Another possible explanation for sex biased phenotypes in *caper* mutant animals may be aberrant dosage compensation, since one of the GO Terms enriched from Caper RNA targets is for dosage compensation. The proteins Msl-1, Msl-2, Msl-3, Mle, and Mof have been demonstrated to form complexes that are important for binding the X-chromosome in males (Scott et al., 2000). Furthermore, loss-of-function mutations in any of these genes in males results in increased lethality. Additionally, the X-linked non-coding gene *roX1*, also appears to be involved in the formation of the dosage compensation complex (Scott et al., 2000; Hallacli et al., 2012; Tikhonova et al., 2019). Considering Caper targets *msl-1, msl-3, mle, mof*, and *roX1*, it suggests that *caper* dysfunction could result in downstream dosage compensation effects. It is also important to note that Mle is a protein interactor of Caper. However, since no other proteins involved in dosage compensation co-immunoprecipitate with Caper, it is unlikely Caper is directly involved in the formation of the dosage compensation complex. Finally, the gene *over compensating males* (*ocm*) encodes an mRNA targeted by Caper that has also been implicated in dosage compensation. *Ocm* appears to work antagonistically to the dosage compensation complex in *Drosophila* and reducing the expression of *ocm* results in rescue of lethality observed in flies with reduced function of *msl-1*. Conversely, reduction of *ocm* results in female sterility (Lim and Kelley, 2013).

### A candidate-based modifier screen of gravitaxis phenotypes

We took advantage of the well-established assay to perform a candidate-based screen for genetic modifiers of *caper* in gravitaxis behavior and to independently verify a subset of Caper interacting proteins and RNAs. Thirteen genes were identified in the gravitaxis screen:*, pur-alpha, sna, cd, superdeath, qkr58E-1*, *t, dab, syp, numb, repo, dco, Acn, CRMP.* Unsurprisingly, most of these genes function in the *Drosophila* nervous system, where they are involved in the development, maintenance, and function of the nervous system. For example, Numb, a protein interactor of Caper, is involved in sensory organ precursor (SOP) asymmetric division and in the chordotonal (ch) cell lineage (Uemura et al., 1989; Rhyu et al., 1994). Since SOPs and ch organs are important for gravity perception, it is unsurprising that *numb* is one of the strongest enhancers of the *caper* gravitaxis phenotype.

While Numb is important for neuronal differentiation, *repo* is indispensable for glial cells. *repo,* an RNA target of Caper, is a glial-specific homeodomain transcription factor essential for the migration, differentiation, and maintenance of glial cells in *Drosophila* (Xiong et al., 1994; Halter et al., 1995) and for long-term memory formation (Matsuno et al., 2015). *repo* mutations result in neuronal cell death, aberrant neuronal morphology, and loss of glial cells. Though Repo does not appear to function during initial glial fate determination, it is important for late glial differentiation (Jones, 2005). Since Caper targets *repo* RNA, but *repo* has only one annotated isoform ruling out a role for Caper in splicing *repo* pre-mRNA, it is possible that Caper regulates its translation.

Additionally, interactors of *caper* play an integral role in neurite morphogenesis and maintenance. Though *CRMP* is not an RNA target of Caper nor encodes a protein interactor, it is a modifier of the *caper* gravitaxis, longevity, and bristle patterning phenotypes (unpublished data). There are five members of the CRMP family of proteins in humans and their phosphorylation influences their biological function (Nakamura et al., 2020). For example, non-phosphorylated CRMP2 promotes axonal elongation. In contrast, phosphorylated CRMP2 inhibits axonal guidance. Additionally, phosphorylation/dephosphorylation of CRMP family members affect CNS degeneration and regeneration. CRMPs are involved in neurite outgrowth, axon guidance, dendritic branching, and synapse maturation in mammals (Nakamura et al., 2020). In addition to CRMP, Dab functions in axonogenesis. Dab, a protein interactor of Caper is an evolutionarily conserved adaptor protein that physically interacts and functions with Able (Abl), a protein tyrosine kinase, during *Drosophila* embryonic axonogenesis (Gertler et al., 1993; Song et al., 2010; Kannan et al., 2017).

### Caper genetic interactors are implicated in NMJ morphogenesis

Since *caper* dysfunction results in aberrant NMJ morphology, it is particularly interesting that several genetic interactors of *caper* are also involved in the development of the NMJ. Syp, a protein interactor of Caper regulates synaptic growth and plasticity in the *Drosophila* larval NMJ by regulating the translation of key mRNAs with important synaptic functions by associating with RNA encoding key proteins involved in synaptic function, such as *futsch, discs large, alpha-spectrin, msp-300, syd-1, highwire,* and *neurexin-1* (McDermott et al., 2014). Additionally, Syp is required for proper NMJ morphology. Overexpression of Syp in muscle suppresses NMJ growth, while loss of Syp function results in overelaboration of the NMJ, a phenotype similar to *caper* mutant NMJs (McDermott et al., 2014; Titus et al., 2021). *Acn* also regulates NMJ morphology (Laviolette et al., 2005). Since *caper* mutants exhibit aberrant NMJ morphology (Titus et al., 2021), it will be interesting to see if *syp* and *Acn* can also modify *caper* NMJ phenotypes.

In sum, our identification of Caper interacting proteins and RNAs in combination with a genetic modifier screen of a subset of these interactors provides better context of the role *caper* plays in the development, maintenance and function of the nervous system. Furthermore, we find that Caper interacts with distinct proteins in neuronal tissue compared to muscles, which begins to illuminate why the nervous system may be more sensitive to the dysfunction of ubiquitously expressed RBPs.

## Data availability statement

The original contributions presented in the study have been deposited in the GEO database. This data can be found at: https://www.ncbi.nlm.nih.gov/geo/query/acc.cgi?acc=GSE221381 accession number GSE221381.

## Author contributions

MT and AC performed the experiments and wrote the manuscript. CE performed the mass spectrometry. NP performed the RNA sequencing analysis. JB performed the statistical analysis. EO conceptualized and designed experiments, obtained funding and resources, and supervised the project. All authors contributed to the article and approved the submitted version.

## Funding

This work was supported by the National Institutes of Health 1R15NS104976 to EO and NINDS-NS080685 to MT. The proteomics works was supported by the National Institutes of Health S10-OD025267 to CE.

## Conflict of interest

The authors declare that the research was conducted in the absence of any commercial or financial relationships that could be construed as a potential conflict of interest.

## Publisher’s note

All claims expressed in this article are solely those of the authors and do not necessarily represent those of their affiliated organizations, or those of the publisher, the editors and the reviewers. Any product that may be evaluated in this article, or claim that may be made by its manufacturer, is not guaranteed or endorsed by the publisher.

## Footnotes

^1^ Bioconductor.org

## Supplementary material

Supplementary Figure S1 Twelve RNA targets identified by RIP-Seq were verified by RT-PCR. RT-PCR of RNA isolated from immunoprecipates and their respective inputs shows that the following genes are enriched in the FLAG IPs relative to the mock IP controls: *qkr58E-1*, *chic*, *unc-115a*, *pros*, *lola*, *zfh1*, *Acn*, *unc-115b*, *dlg1*, *tutl*, *brp*, and *chinmo*.
