# Supplementary material for: The identification of protein and RNA interactors of the splicing factor Caper in the adult Drosophila nervous system
Source: Front Mol Neurosci. 2023 Jun 23;16:1114857. doi: 10.3389/fnmol.2023.1114857 (PMC10332324; doi:10.3389/fnmol.2023.1114857)
Supplement: Supplementary file 12 [file Table_12.docx]

| **Transheterozygote** | **Factor** | **χ^2^** | **P value** | **vs. control** | **Sex** | **t ratio** | **P value** |
| --- | --- | --- | --- | --- | --- | --- | --- |
| *caper^-/+^;CRMP[supla1]^-/+^* | Genotype | 16.7 | 0.0002 | *yw^-/+^;caper^-/+^* | F | 4.6 | 8.1E-5 |
|  | Sex | 75.7 | 3.3E-18 | *yw^-/+^;CRMP[supla1]^-/+^* | F | 3.8 | 0.0018 |
|  | Genotype x Sex | 8.2 | 0.0161 | *yw^-/+^;caper^-/+^* | M | 0.7 | 1 |
|  |  |  |  | *yw^-/+^;CRMP[supla1]^-/+^* | M | -1.2 | 1 |
| *caper^-/+^;CRMP[supK1]^-/+^* | Genotype | 39.7 | 2.3E-9 | *yw^-/+^;caper^-/+^* | F | 6.8 | 9.5E-10 |
|  | Sex | 109.3 | 1.3E-25 | *caper^-/+^;CRMP[supK1]^-/+^* | F | 3.9 | 0.0013 |
|  | Genotype x Sex | 39.7 | 2.3E-9 | *yw^-/+^;caper^-/+^* | M | 1.4 | 1 |
|  |  |  |  | *caper^-/+^;CRMP[supK1]^-/+^* | M | -1.4 | 1 |
| *caper^-/+^; repo^lacz/+^* | Genotype | 23.9 | 6.3E-6 | *yw^-/+^;caper^-/+^* | F | 5.0 | 2.8E-5 |
|  | Sex | 74.0 | 7.9E-18 | *yw^-/+^;repo^lacz/+^* | F | 3.0 | 0.0322 |
|  | Genotype x Sex | 7.0 | 0.0307 | *yw^-/+^;caper^-/+^* | M | 2.4 | 0.1841 |
|  |  |  |  | *yw^-/+^;repo^lacz/+^* | M | 2.5 | 0.1234 |
| *caper^-/+^; superdeath^-/+^* | Genotype | 16.0 | 0.0003 | *yw^-/+^;caper^-/+^* | F | 4.2 | 0.0004 |
|  | Sex | 94.6 | 2.3E-22 | *yw^-/+^;superdeath^-/+^* | F | 5.1 | 1.4E-5 |
|  | Genotype x Sex | 16.6 | 0.0002 | *yw^-/+^;caper^-/+^* | M | -0.73 | 1 |
|  |  |  |  | *yw^-/+^;superdeath^-/+^* | M | -1.9 | 0.4919 |
| *caper^-/+^; puralpha^-/+^* | Genotype | 31.3 | 1.6E-7 | *yw^-/+^;caper^-/+^* | F | 2.9 | 0.0380 |
|  | Sex | 87.1 | 1.0E-20 | *yw^-/+^;puralpha^-/+^* | F | -3.1 | 0.0206 |
|  | Genotype x Sex | 8.2 | 0.0165 | *yw^-/+^;caper^-/+^* | M | 3.0 | 0.0282 |
|  |  |  |  | *yw^-/+^;puralpha^-/+^* | M | -0.8 | 1 |
| *caper^-/+^; tan1^-/+^* | Genotype | 66.3 | 3.9E-15 | *yw^-/+^;caper^-/+^* | F | 2.9 | 0.0407 |
|  | Sex | 23.6 | 1.2E-6 | *yw^-/+^;tan1^-/+^* | F | -3.7 | 0.0027 |
|  | Genotype x Sex | 8.6 | 0.0136 | *yw^-/+^;caper^-/+^* | M | 7.3 | 4.7E-10 |
|  |  |  |  | *yw^-/+^;tan^-/+^* | M | 0.0 | 1 |
| *caper^-/+^; cd1^-/+^* | Genotype | 14.8 | 0.0006 | *yw^-/+^;caper^-/+^* | F | 3.5 | 0.0062 |
|  | Sex | 104.3 | 1.8E-24 | *yw^-/+^;cd1^-/+^* | F | -3.3 | 0.0132 |
|  | Genotye x Sex | 28.5 | 6.5E-7 | *yw^-/+^;caper^-/+^* | M | -0.3 | 1 |
|  |  |  |  | *yw^-/+^;cd1^-/+^* | M | 0.2 | 1 |

**Table S12.** Results of log-logistic survival analysis and *post-hoc* comparisons of estimated marginal means using Tukey’s method for models with a significant genotype x sex interaction.
