# Supplementary material for: The identification of protein and RNA interactors of the splicing factor Caper in the adult Drosophila nervous system
Source: Front Mol Neurosci. 2023 Jun 23;16:1114857. doi: 10.3389/fnmol.2023.1114857 (PMC10332324; doi:10.3389/fnmol.2023.1114857)
Supplement: Supplementary file 13 [file Table_13.docx]

| **Transheterozygote** | **Factor** | **χ^2^** | **P value** | **vs. control** | **t ratio** | **P value** |
| --- | --- | --- | --- | --- | --- | --- |
| *caper^-/+^; Acn^FS1/+^* | Genotype | 19.7 | 5.7E-05 | *yw^-/+^;caper^-/+^* | 4.4 | 6.4E-05 |
|  | Sex | 152.5 | 5.0E-35 | *yw^-/+^;Acn^FS1/+^* | 2.9 | 0.0146 |
|  | Genotype x Sex | 2.3 | 0.3107 |  |  |  |
| *caper^-/+^; dab^2/+^* | Genotype | 25.9 | 2.3E-06 | *yw^-/+^;caper^-/+^* | 2.6 | 0.0311 |
|  | Sex | 54.2 | 1.8E-13 | *yw^-/+^;dab^2/+^* | 5.3 | 1.8E-06 |
|  | Genotype x Sex | 5.8 | 0.0550 |  |  |  |
| *caper^-/+^; dco^80977/+^* | Genotype | 38.7 | 4.0E-09 | *yw^-/+^;caper^-/+^* | 6.1 | 4.8E-08 |
|  | Sex | 25.6 | 4.2E-07 | *yw^-/+^;dco^80977/+^* | 5.6 | 4.4E-07 |
|  | Genotype x Sex | 2.1 | 0.3483 |  |  |  |
| *caper^-/+^; numb^-/+^* | Genotype | 67.0 | 2.9E-15 | *yw^-/+^;caper^-/+^* | 9.5 | 6.6E-16 |
|  | Sex | 86.2 | 1.6E-20 | *yw^-/+^;numb^/+^* | 3.8 | 0.0007 |
|  | Genotype x Sex | 12.7 | 0.0017 |  |  |  |
| *caper^-/+^; qkr58E-1^-/+^* | Genotype | 6.9 | 0.0314 | *yw^-/+^;caper^-/+^* | 2.6 | 0.0346 |
|  | Sex | 49.0 | 2.6E-12 | *yw^-/+^;qkr1^-/+^* | 2.5 | 0.0462 |
|  | Genotype x Sex | 2.8 | 0.2524 |  |  |  |
| *caper^-/+^; sna^-/+^* | Genotype | 28.2 | 7.5E-07 | *yw^-/+^;caper^-/+^* | 5.7 | 4.0E-07 |
|  | Sex | 31.2 | 2.4E-08 | *yw^-/+^;sna^-/+^* | 2.6 | 0.0337 |
|  | Genotype x Sex | 2.1 | 0.3565 |  |  |  |
| *caper^-/+^; syp^55577/+^* | Genotype | 11.6 | 0.0030 | *yw^-/+^;caper^-/+^* | 3.1 | 0.0062 |
|  | Sex | 74.6 | 5.8E-18 | *yw^-/+^;syp^55577/+^* | 3.1 | 0.0059 |
|  | Genotype x Sex | 2.2 | 0.3326 |  |  |  |

**Table S13.** Results of log-logistic survival analysis and *post-hoc* comparisons of estimated marginal means using Tukey’s method for models with a significant genotype effect. Although *numb* showed a significant interaction, the transheterozygote was only different from one of the controls so the main genotype effect is reported instead.
