# Supplementary figures and images for: The identification of protein and RNA interactors of the splicing factor Caper in the adult Drosophila nervous system
Source: Front Mol Neurosci. 2023 Jun 23;16:1114857. doi: 10.3389/fnmol.2023.1114857 (PMC10332324; doi:10.3389/fnmol.2023.1114857)

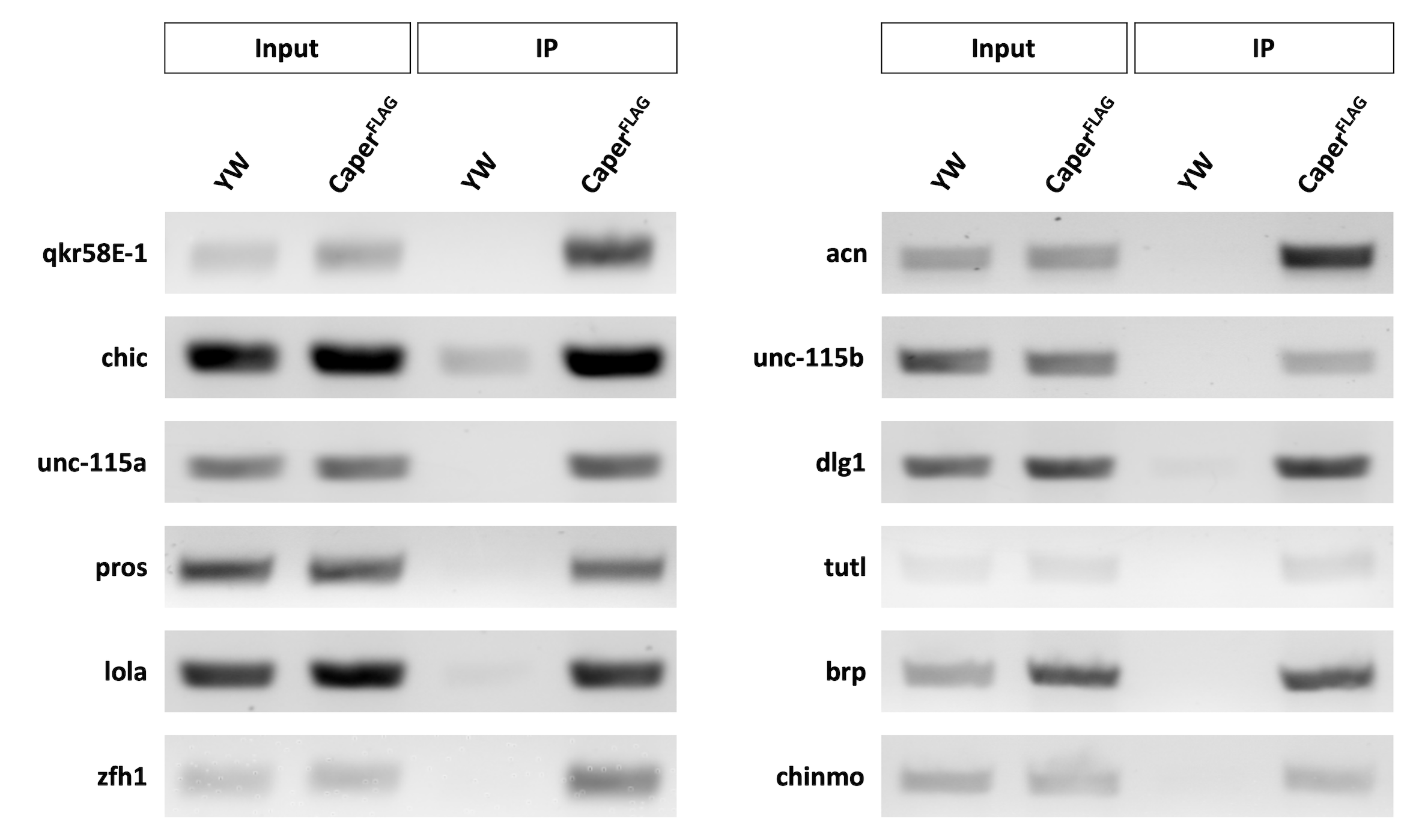

Supplement: Supplementary Figure S1 — Twelve RNA targets identified by RIP-Seq were verified by RT-PCR. RT-PCR of RNA isolated from immunoprecipates and their respective inputs shows that the following genes are enriched in the FLAG IPs relative to the mock IP controls: qkr58E-1, chic, unc-115a, pros, lola, zfh1, Acn, unc-115b, dlg1, tutl, brp, and chinmo. [file Image_1.TIFF]
